# Development and validation of nomogram to predict very early recurrence of combined hepatocellular-cholangiocarcinoma after hepatic resection: a multi-institutional study

**DOI:** 10.1186/s12957-022-02536-y

**Published:** 2022-02-28

**Authors:** Yijun Wu, Hongzhi Liu, Jianxing Zeng, Yifan Chen, Guoxu Fang, Jinyu Zhang, Weiping Zhou, Yongyi Zeng, Jingfeng Liu

**Affiliations:** 1 Department of Hepatobiliary Surgery, Mengchao Hepatobiliary Hospital of Fujian Medical University, Fuzhou, 350025 People’s Republic of China; 2 Shengli Clinical Medical College of Fujian Medical University, Fuzhoum, People’s Republic of China; 3 Department of Hepatobiliary Surgery, Eastern Hepatobiliary Surgery Hospital, Second Military Medical University, Shanghai, 200438 People’s Republic of China; 4 Fujian Medical University Cancer Hospital & Fujian Cancer Hospital, No. 420, Fuma Road, Fuzhou, 350014 Fujian People’s Republic of China; 5 The Big Data Institute of Southeast Hepatobiliary Health Information, Mengchao Hepatobiliary Hospital of Fujian Medical University, Fuzhou, 350025 People’s Republic of China

**Keywords:** Combined hepatocellular-cholangiocarcinoma (cHCC), Very early recurrence, Nomogram, Prognosis

## Abstract

**Background and objectives:**

Combined hepatocellular cholangiocarcinoma (cHCC) has a high incidence of early recurrence. The objective of this study is to construct a model predicting very early recurrence (VER) (i.e., recurrence within 6 months after surgery) of cHCC.

**Methods:**

One hundred thirty-one consecutive patients from Eastern Hepatobiliary Surgery Hospital served as a development cohort to construct a nomogram predicting VER by using multi-variable logistic regression analysis. The model was internally and externally validated in a validation cohort of 90 patients from Mengchao Hepatobiliary Hospital using the C concordance statistic, calibration analysis, and decision curve analysis (DCA).

**Results:**

The VER nomogram contains microvascular invasion (MiVI), macrovascular invasion (MaVI), and CA19-9 > 25 mAU/mL. The model shows good discrimination with C-indexes of 0.77 (95% CI: 0.69–0.85) and 0.76 (95% CI: 0.66–0.86) in the development cohort and validation cohort respectively. Decision curve analysis demonstrated that the model is clinically useful and the calibration of our model was favorable. Our model stratified patients into two different risk groups, which exhibited significantly different VER.

**Conclusions:**

Our model demonstrated favorable performance in predicting VER in cHCC patients.

## Introduction

Combined hepatocellular cholangiocarcinoma (cHCC) is a rare subtype, accounting for only 0.4–14.2% of primary liver carcinoma (PLC) [1–5]. Due to its rarity and a wide variety of pathological types, the research in cHCC has been tough and the clinicopathological characteristics and prognosis of cHCC still remain poorly understood.

It is well-acknowledged that the prognosis of cHCC is dismal, due to its high recurrence rate after hepatic resection [2]. On the basis of previous studies [1–5], most recurrences occur early after the hepatic resection for cHCC. Patients with cHCC suffered early recurrence (ER) rate of 57–75% and 6 months recurrence rate of about 40% or so [4–9], higher than patients with HCC, similar to patients with ICC [2, 10].

Nevertheless, most studies only concentrated on ER for patients with cHCC using a cut-off of 2 years, which consists with the cut-off for ER of hepatocellular carcinoma (HCC) and intrahepatic cholangiocarcinoma (ICC) [4–8]. The ER rate and the relationship between ER and clinicopathological characteristics of cHCC have been researched in these studies [4–8]. Given that patients with cHCC suffered earlier recurrence and a higher rate of recurrence than patients with HCC [2, 3], it may not be appropriate to use a cut-off of 2 years to differentiate early and late recurrence for patients with cHCC or ICC [11], aligning with patients with HCC.

In the field of other malignant tumors [11–13], including HCC and ICC [11, 13], researchers have noticed that patients who had VER(i.e., recurrence within 6 months after surgery) suffered a significant lower OS than those who did not have VER, and therefore brought forward the definition of VER and investigated the risk factors of VER. The research on VER would be of significance for patients with cHCC. As such, the tool we developed to predict VER in patients with cHCC after hepatic resection would help identify the patients at high risk for VER, therefore making a contribution to constructing individualized surveillance strategies following hepatic resection or recommending treatment postoperatively.

To the best of our knowledge, no study exists has investigated the prediction of VER of cHCC after surgical resection. Therefore, our study aims to illustrate the relationship between the clinicopathological characteristics and VER, develop, and validate a model predicting VER after hepatic resection for cHCC based on multi-institutional data sets. In addition, we have constructed an online calculator to promote the use of our model in clinical work (https://chcrecurrence.shinyapps.io/myCHCVER2).

## Patients and methods

The analysis was presented in accordance with the TRIPOD (Transparent reporting of a multi-variable prediction model for individual prognosis or diagnosis) guidelines [14].

### Patients

In this retrospective multi-institutional study, a total of 221 patients received hepatic resection for cHCC between January 2011 and December 2015 were enrolled from 2 institutions, compromising Eastern Hepatobiliary Surgery Hospital of Second Military Medical University (Institution I) and Mengchao Hepatobiliary Hospital of Fujian Medical University (Institution II). This study was conducted in accordance with the ethical guideline of the 1975 Declaration of Helsinki and obtained approval from the Institutional Ethics Committee of the Mengchao Hepatobiliary Hospital of Fujian Medical University. Informed consent has been given to each participants enrolled in this study.

The inclusion criteria include (1) patients underwent curative hepatic resection for primary cHCC and pathologically confirmed as cHCC (only Allen and Lisa classification type C ); (2) Child-Pugh A or B liver function; (3) no previous anti-tumor treatment; (4) no extrahepatic metastasis; and (5) R0 resection, defined as complete removal of macroscopic tumor nodules with a clear margin. The exclusion criteria include (1) other malignant tumors; (2) incomplete clinical data; and (3) loss to follow-up within 12 months after the surgery.

**Fig. 1:**
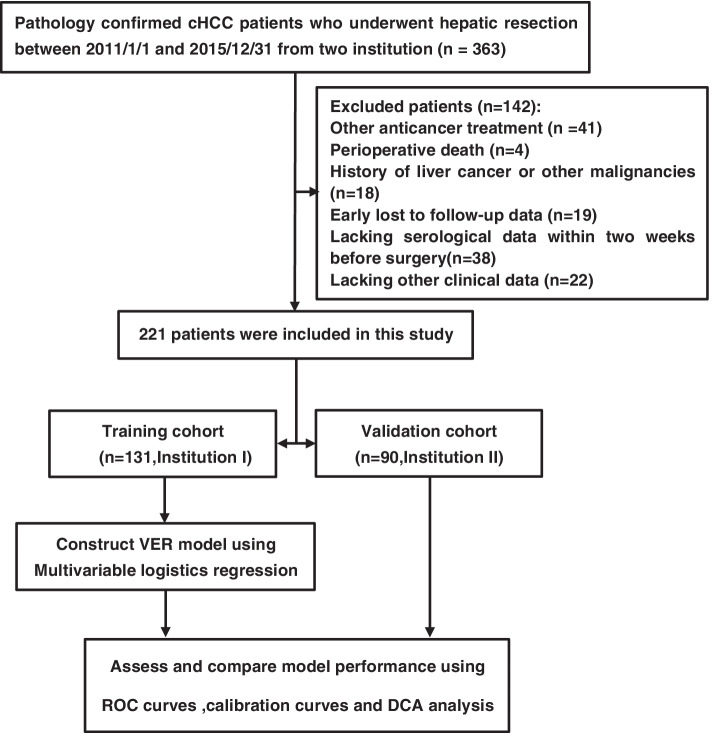
The flow chart of this study

One hundred thirty-one eligible patients form Institution I were assigned to development cohort for the construction of the predictive model and 90 eligible patients form Institution II were allocated to validation cohort for the verification of the models. The flow chart of this study was shown in Fig. 1.

### Hepatic resection

All patients underwent hepatic resection, the type of which was determined by the tumor size, tumor location, preoperative diagnosis, and liver function. Hepatic resection without lymph node dissection was performed to the patients who were diagnosed as HCC preoperatively. Hepatic resection with lymph node dissection was performed to the patients who were diagnosed as ICC or cHCC preoperatively. Lymph node dissection was performed when an abnormal swollen lymph node around the hepatoduodenal ligament, the common hepatic artery, or behind the pancreas head was seen intraoperatively. Major resection was defined as resection of 3 or more Couinaud segments, while minor resection was defined as resection of fewer than 3 Couinaud segments [15].

### Clinicopathological characteristics and definitions

The clinicopathological variables were listed as follows: gender, age, hepatitis B virus (HBV), hepatitis C virus (HBV), non-alcoholic fatty liver disease(NAFLD), red blood cell count, hemoglobin, platelet count, neutrophil count, lymphocyte count, neutrophils/lymphocytes ratio , aspartate aminotransferase, prothrombin time, total bilirubin, albumin, gamma-glutamyl transpeptidase, alkaline phosphatase, alpha-fetoprotein, carbohydrate antigen 19-9 level, carcinoembryonic antigen, decarboxylic prothrombin, liver cirrhosis, resection type, lymph node dissection, tumor number, maximum tumor size, tumor capsule, microvascular invasion (MiVI), macrovascular invasion(MaVI), satellite nodules (SN), Edmondson-Steiner classification [16], intraoperative blood loss, intraoperative blood transfusion, Child-Pugh classification, and AJCC 8th edition staging manual. We collected the serological examinations carried out within 2 weeks before the surgery. Two independent pathologists majored in hepatic tumors reviewed the resected specimens. MiVI was defined as the presence of tumor cell nests in the portal vein, the hepatic vein or the capsular vessel lined by endothelium visible only under microscopy [17], MaVI was defined as invasion of the first-and second- order branches of the portal veins, hepatic arteries or hepatic veins [18], SN was defined as tumor cell nests present on microscopy or tumors of which the maximum diameter is less than 2 cm resenting within 2 cm of the main tumor on macroscopy [19]. Tumor stage was defined in accordance with the AJCC 8th edition ICC staging manual [20].

### Follow-up

After the surgery, patients were followed up every 3 months in the first 2 years and every 6–12 months thereafter. Serum tumor markers, contrast-enhanced magnetic resonance imaging (MRI) of abdomen were performed to diagnose the recurrence of cHCC. Patients with recurrence of cHCC received appropriate treatments including transarterial chemoembolization (TACE), radiofrequency ablation or hepatic re-resection. As the primary end point, recurrence-free survival (RFS) was defined as the interval from hepatic resection to recurrence. VER of cHCC was defined as recurrence within 6 months after surgery on the basis of previous studies [11–13, 21]. We collected the survival information until March 31, 2020.

### Statistical analysis

Categorical variables were presented as number (percentage) and compared using the chi-square test or Fisher exact test. Normally distributed continuous variables were presented as mean (standard deviation, IQR) and compared using Mann-Whitney *U* test. The *P* value less than 0.05 was set as statistical significance in this study. Statistical analysis was conducted by using R version 4.1.0 (http://www.r-project.org/) with R packages of Table 1, rms, pROC, ggplot2, shiny, survminer, and survival.

**Table 1:** The clinicopathologic characteristics of patients in the development cohort and the validation cohort

Variables	Development cohort (*n* = 131)	Validation cohort (*n* = 90)	*P* value
Gender, male, *n* (%)	117 (89.3%)	70 (77.8%)	0.032
Age [year, mean (IQR)]	52.0 [46.0, 60.5]	52.5 [45.0, 63.0]	0.541
Hepatitis			
NBNC, *n* (%)	16 (12.2%)	14 (15.6%)	0.756
HBV, *n* (%)	113 (86.3%)	75 (83.3%)	
HCV, *n* (%)	2 (1.5%)	1 (1.1%)	
NAFLD, *n* (%)	5 (3.8%)	6 (6.7%)	0.521
Cirrhosis, *n* (%)	80.0 (61.1%)	69.0 (76.7%)	0.022
Resection type			
Minor resection, *n* (%)	44 (33.6%)	34 (37.8%)	0.619
Major resection, *n* (%)	87 (66.4%)	56 (62.2%)	
Lymph node dissection, *n* (%)	19 (14.5%)	21 (23.3%)	0.134
Tumor number, multiple, *n* (%)	34 (26.0%)	25 (27.8%)	0.884
Tumor size [cm, mean (IQR)]	5.10 [3.60, 7.35]	5.30 [3.03, 9.00]	0.737
Tumor capsule, present, *n* (%)	85 (64.9%)	39 (43.3%)	0.002
Microvascular invasion, *n* (%)	77 (58.8%)	64 (71.1%)	0.083
Macrovascular invasion, *n* (%)	23 (17.6%)	31 (34.4%)	0.007
Satellite nodules, *n* (%)	48 (36.6%)	28 (31.1%)	0.480
Edmondson-Steiner classification			
I/II, *n* (%)	107 (81.7%)	67 (74.4%)	0.261
III/IV, *n* (%)	24 (18.3%)	23 (25.6%)	
RBC[10^12/L, mean (IQR)]	4.63 [4.40, 4.92]	4.74 [4.29, 5.12]	0.615
Hb[g/L, mean (IQR)]	143 [133, 150]	145 [132, 155]	0.781
PLT[10^9/L, mean (IQR)]	175 [127, 235]	180 [126, 216]	0.994
Neutrophil count[10^9/L, mean (IQR)]	3.77 [3.09, 4.76]	3.97 [2.85, 5.36]	0.116
Lymphocyte count [10^9/L, mean (IQR)]	1.59 [1.16, 1.92]	1.42 [1.15, 1.83]	0.705
NLR[ratio, mean (IQR)]	2.42 [1.74, 3.45]	2.53 [1.66, 3.78]	0.227
AST[U/L, mean (IQR)]	39.4 [39.4, 126]	34.5 [25.0, 58.0]	0.133
PT[s, mean (IQR)]	11.5 [11.0, 12.1]	13.4 [12.8, 14.1]	< 0.001
TB[umol/L, mean (IQR)]	13.3 [10.5, 18.1]	7.00 [3.80, 12.1]	0.870
ALB[g/L, mean (IQR)]	41.4 [39.0, 44.5]	39.5 [36.0, 42.8]	< 0.001
GGT[U/L, mean (IQR)]	66.0 [33.5, 138]	65.0 [42.0, 128]	0.171
ALP[U/L, mean (IQR)]	87.0 [69.5, 118]	94.0 [74.3, 117]	0.339
AFP[ng/mL, mean (IQR)]	51.0 [7.95, 373]	76.5 [9.13, 1540]	0.250
CEA[ng/mL, mean (IQR)]	2.50 [1.70, 3.70]	2.86 [1.70, 4.40]	0.725
CA19-9[U/mL, mean (IQR)]	27.7 [13.8, 71.3]	20.4 [10.2, 44.8]	0.986
DCP[mAU/mL, mean (IQR)]	64.0 [23.0, 412]	110 [40.0, 940]	0.057
Intraoperative blood loss [mL, mean (IQR)]	200 [100, 300]	200 [100, 288]	0.105
Intraoperative blood transfusion, *n* (%)	37 (28.2%)	33 (36.7%)	0.240
Child-Pugh			
A, *n* (%)	130 (99.2%)	83 (92.2%)	0.018
B, *n* (%)	1 (0.8%)	7 (7.8%)	
AJCC 8th			
IA-II, *n* (%)	111 (84.7%)	71 (78.9%)	0.347
III-IV, *n* (%)	20 (15.3%)	19 (21.1%)	

Continuous variables were presented as number (percentage) and compared using the chi-square test or Fisher exact test. Normally distributed continuous variables were presented as mean (standard deviation, IQR). *HBV* hepatitis B virus, *HCV* hepatitis C virus, *NAFLD* non-alcoholic fatty liver disease, *RBC* red blood cell, *Hb* hemoglobin, *PLT* platelet count, *NLR* neutrophils/lymphocytes ratio, *AST* aspartate aminotransferase, *PT* prothrombin time, *TB* total bilirubin, *ALB* albumin, *GGT* gamma-glutamyl transpeptidase, *ALP* alkaline phosphatase, *AFP* alpha-fetoprotein, *CEA* carcinoembryonic antigen, *CA19-9* carbohydrate antigen 19-9, *DCP* decarboxylic prothrombin, *AJCC* American Joint Committee on Cancer

The optimal thresholds of continuous variables were determined by analyzing the ROC curves and Youden index. Clinicopathological variables showing potentially relevance (with *p* < 0.05 in the univariable logistic regression analysis) were employed for the multi-variable logistic regression analysis. The prognostic factors determined by using a stepwise selection method were integrated to construct the VER model. Nomogram and an online calculator are built to facilitate the clinical use of our model [22] (https://chcrecurrence.shinyapps.io/myCHCVER2). The VER risk scores of patients were calculated in accordance with the VER model, then the probabilities of VER were estimated by using the formula: VER probability= 1/{1+exp[−(VER risk score)]}. All patients were stratified into two risk groups depending on their VER probability by the cut-offs of 50th centile. Kaplan-Meier curves and log-rank test were employed to evaluate the difference in RFS between different subgroups.

To assess the predictive accuracy of our model, we drew the ROC curves and calculated the C-indexes of our model and AJCC staging for the development cohort and validation cohort with the bootstrapping resample method (*n* = 1000). We conducted the decision curve analysis(DCA) to estimate the clinical net benefits of our model and AJCC staging for both cohorts with the bootstrapping resample method (*n* = 1000) [23]. Calibration curves were employed to plot the predicted probabilities verses the actual outcomes.

## Results

### Clinicopathologic characteristics

A total of 363 cHCC patients underwent hepatic section in Eastern Hepatobiliary Surgery Hospital of Second Military Medical University (Institution I) and Mengchao Hepatobiliary Hospital of Fujian Medical University (Institution II) between 1 January 2011 and 31 December 2015 received careful reviews of their medical records, 221 patients were eligible for the study, 142 patients were excluded for receiving other anti-cancer treatment (*n* = 41), perioperative death (*n* = 4), history of liver cancer or other malignancies (*n* = 18), early lost to follow-up data (*n* = 19), lacking serological data within 2 weeks before surgery (*n* = 38), lacking other clinical data (*n* = 22). One hundred thirty-one eligible patients from Institution I were enrolled as the development cohort and 90 eligible patients from Institution II serve as the validation cohort. The flow chart of this study is shown in Fig. 1.

The clinicopathologic characteristics of patients in the development cohort and the validation cohort are summarized in Table 1. Variables including gender, cirrhosis, tumor capsule, PT, ALB, and Child-Pugh show differences between the two institutions. In the overall cohort, the development cohort and the validation cohort, respectively, 136 patients (61.5%), 82 patients (62.6%), and 54 patients (60%) had early recurrence (< 2 years); 96 patients (43.4%), 54 patients (41.2%), and 42 patients (46.7%) had 6-month VER; 121 patients (54.8%), 72 patients (60.0%), and 49 patients (54.4%) had 12-month recurrence.

The comparison of clinicopathologic characteristics between patients with and without VER is summarized in Table 2. We found that people developed VER tend to have these clinical factors: the performance of major resection, lymph node dissection, tumor size> = 5 cm, the presence of MiVI, MaVI, and SN, GGT> = 75 U/L, ALP > = 78 U/L, AFP > = 20 ng/mL, CA19-9 > = 25 U/mL, and DCP > = 200 mAU/mL.

**Table 2:** Comparison of clinicopathologic characteristics between patients with and without VER

Variables	VER (*n* = 96)	Non-VER (*n* = 125)	*P* value
Gender(male)	79 (82.3%)	108 (86.4%)	0.515
Age (>= 65 years)	15 (15.6%)	24 (19.2%)	0.608
Hepatitis			
NBNC, *n* (%)	12 (12.5%)	18 (14.4%)	0.668
HBV, *n* (%)	82 (85.4%)	106 (84.8%)	
HCV, *n* (%)	2 (2.1%)	1 (0.8%)	
NAFLD, *n* (%)	5 (5.2%)	6 (4.8%)	1
Cirrhosis (yes)	65 (67.7%)	84 (67.2%)	1
Resection type (major resection)	70 (72.9%)	73 (58.4%)	0.036
Lymph node dissection (yes)	24 (25.0%)	16 (12.8%)	0.031
Tumor number (multiple)	30 (31.3%)	29 (23.2%)	0.235
Tumor size (>= 5 cm)	66 (68.8%)	55 (44.0%)	< 0.001
Tumor capsule (present)	52 (54.2%)	72 (57.6%)	0.709
Microvascular invasion (present)	77 (80.2%)	64 (51.2%)	< 0.001
Macrovascular invasion (present)	38 (39.6%)	16 (12.8%)	< 0.001
Satellite nodules (present)	48 (50.0%)	28 (22.4%)	< 0.001
Edmondson-Steiner classification (III/IV)	21 (21.9%)	26 (20.8%)	0.978
RBC(>= 4 × 10^12/L)	10 (10.4%)	13 (10.4%)	1
Hb(< 120*g/L)	7 (7.3%)	9 (7.2%)	1
PLT(< 300 × 10^9/L)	86 (89.6%)	117 (93.6%)	0.404
Neutrophil count(>= 5 × 10^9/L)	63 (65.6%)	97 (77.6%)	0.068
Lymphocyte count(>= 1.5 × 10^9/L)	23 (24.0%)	32 (25.6%)	0.902
NLR(> = 1.6)	18 (18.8%)	30 (24.0%)	0.439
AST(>= 40 U/L)	49 (51.0%)	56 (44.8%)	0.432
PT(>= 13 s)	35 (36.5%)	40 (32.0%)	0.582
TB(>= 17.1 umol/L)	22 (22.9%)	34 (27.2%)	0.569
ALB(< 40 g/L)	33 (34.4%)	48 (38.4%)	0.635
GGT(>= 75 U/L)	54 (56.3%)	47 (37.6%)	0.009
ALP(>= 78 U/L)	77 (80.2%)	72 (57.6%)	< 0.001
AFP(>= 20 ng/mL)	72 (75.0%)	72 (57.6%)	0.011
CEA(>= 5 ng/mL)	16 (16.7%)	18 (14.4%)	0.783
CA19-9(>= 25 U/mL)	63 (65.6%)	49 (39.2%)	< 0.001
DCP(>= 200 mAU/mL)	41 (42.7%)	32 (25.6%)	0.011
Intraoperative blood loss(>= 200 mL)	60 (62.5%)	63 (50.4%)	0.097
Intraoperative blood transfusion (yes)	28 (29.2%)	42 (33.6%)	0.578

*HBV* hepatitis B virus, *HCV* hepatitis C virus, *NAFLD* non-alcoholic fatty liver disease, *RBC* red blood cell, *Hb* hemoglobin, *PLT* platelet count, *NLR* neutrophils/lymphocytes ratio, *AST* aspartate aminotransferase, *PT* prothrombin time, *TB* total bilirubin, *ALB* albumin, *GGT* gamma-glutamyl transpeptidase, *ALP* alkaline phosphatase, *AFP* alpha-fetoprotein, *CEA* carcinoembryonic antigen, *CA19-9* carbohydrate antigen 19-9, *DCP* decarboxylic prothrombin

### Construction and evaluation of the VER model

**Fig. 2:**
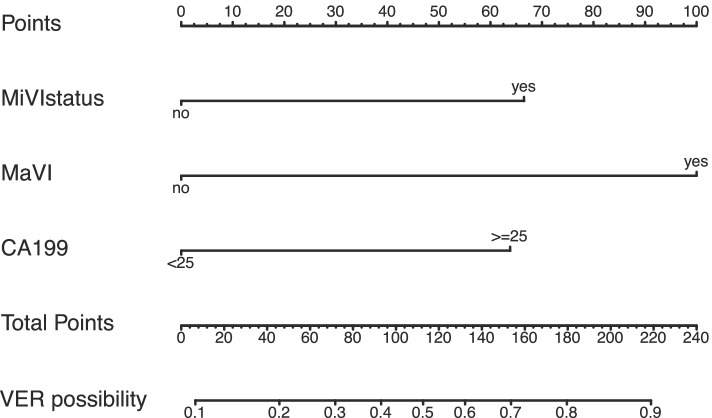
VER nomogram integrating microvascular invasion, macrovascular invasion, and CA19-9> = 25 U/mL

The results of univariable logistic regression analysis and multi-variable logistic regression analysis of VER are listed in Table 3. The independent risk factors of VER including the presence of MiVI (odds ratio 3.73, 95% CI: 1.43–9.71, *P* = 0.007), the presence of MaVI (odds ratio 5.75, 95% CI: 1.73–19.04, *P* = 0.004), and CA19-9 > = 25 U/mL (odds ratio 2.48, 95% CI: 1–6.13, *P* = 0.049) were used to construct the VER model (Table 3). A nomogram integrating the independent risk factors described above was constructed to facilitate the use of our predicting model of the VER (Fig. 2).

**Table 3:** Univariable logistic regression analysis and multivariable logistic regression analysis of VER in development cohort

Variables	Univariable logistic regression analysis		Multivariable logistic regression analysis	
	OR (95% CI)	*P* value	OR (95% CI)	*P* value
Gender(male)	2.83(0.75–10.69)	0.124		
Age (>= 65 years)	0.5(0.17–1.5)	0.219		
Hepatitis				
HBV vs NBNC	1.62(0.53–4.98)	0.396		
HCV vs NBNC	2.2(0.11–42.74)	0.602		
NAFLD (yes)	2.21(0.36–13.67)	0.395		
Cirrhosis (yes)	1(0.49–2.05)	0.993		
Resection type (major resection)	1.82(0.85–3.91)	0.122		
Lymph node dissection (yes)	1.72(0.65–4.56)	0.278		
Tumor number (multiple)	2.65(1.19–5.89)	0.017	1.06(0.36–3.14)	0.920
Tumor size (>= 5 cm)	1.94(0.95–3.94)	0.069		
Tumor capsule (present)	0.76(0.37–1.56)	0.449		
Microvascular invasion (present)	3.09(1.45–6.57)	0.003	3.73(1.43–9.71)	0.007
Macrovascular invasion (present)	5.44(1.98–14.96)	0.001	5.75(1.73–19.04)	0.004
Satellite nodules (present)	3.54(1.68–7.46)	< 0.001	2.5(0.98–6.4)	0.056
Edmondson-Steiner classification(III/IV)	0.66(0.26–1.68)	0.387		
RBC(>= 4 × 10^12/L)	0.8(0.22–2.88)	0.733		
Hb(< 120 × g/L)	1.97(0.42–9.2)	0.387		
PLT(< 300 × 10^9/L)	0.8(0.25–2.53)	0.704		
Neutrophil count(>= 5 × 10^9/L)	0.57(0.26–1.29)	0.181		
Lymphocyte count(>= 1.5 × 10^9/L)	0.97(0.43–2.18)	0.937		
NLR(>= 1.6)	0.57(0.23–1.43)	0.230		
AST(>= 40 U/L)	1(0.5–2.01)	0.991		
PT(>= 13 s)	1.25(0.4–3.95)	0.704		
TB(>= 17.1 umol/L)	0.83(0.39–1.75)	0.618		
ALB(< 40 g/L)	1.03(0.47–2.23)	0.949		
GGT(>= 75 U/L)	1.76(0.87–3.55)	0.116		
ALP(>= 78 U/L)	4.07(1.79–9.23)	< 0.001	2.03(0.77–5.33)	0.152
AFP(>= 20 ng/mL)	2.77(1.28–5.97)	0.009	1.97(0.78–4.96)	0.152
CEA(>= 5 ng/mL)	0.84(0.29–2.46)	0.747		
CA19-9(>= 25 U/mL)	3.66(1.73–7.73)	< 0.001	2.48(1–6.13)	0.049
DCP(>= 200 mAU/mL)	2.62(1.24–5.57)	0.012	1.43(0.57–3.58)	0.440
Intraoperative blood loss(>= 200 mL)	0.96(0.47–1.94)	0.906		
Intraoperative blood transfusion (yes)	0.82(0.38–1.79)	0.622		

*HBV* hepatitis B virus, *HCV* hepatitis C virus, *NAFLD* non-alcoholic fatty liver disease, *RBC* red blood cell, *Hb* hemoglobin, *PLT* platelet count, *NLR* neutrophils/lymphocytes ratio, *AST* aspartate aminotransferase, *PT* prothrombin time, *TB* total bilirubin, *ALB* albumin, *GGT* gamma-glutamyl transpeptidase, *ALP* alkaline phosphatase, *AFP* alpha-fetoprotein, *CEA* carcinoembryonic antigen, *CA19-9* carbohydrate antigen 19-9, *DCP* decarboxylic prothrombin, *CI* confidence interval, *OR* odds ratio

**Fig. 3:**
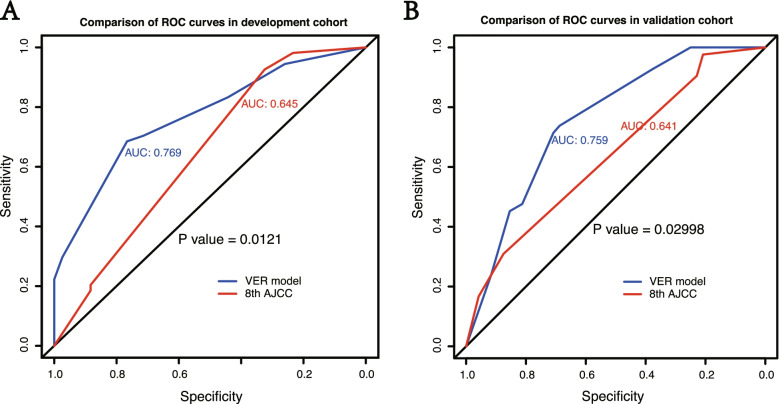
ROC curves for VER model versus 8th AJCC in the development cohort (*P* = 0.012) (**A**) and the validation cohort (*P* = 0.030) (**B**). Our model shows better discrimination with C-indexes of 0.77 (95% CI: 0.69–0.85 ) and 0.76 (95% CI: 0.66–0.86) than AJCC 8th staging with C-indexes of 0.64 (95% CI: 0.57–0.72) and 0.64 (95% CI: 0.54–0.74) in the development cohort and validation cohort, respectively

**Fig. 4:**
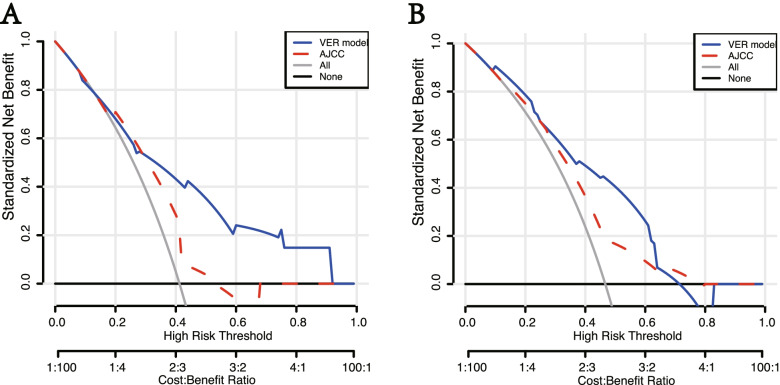
Decision curve analysis for VER model versus 8th AJCC in the development cohort (**A**) and the validation cohort (**B**). Our VER model showed a better net benefit than AJCC 8th staging

**Fig. 5:**
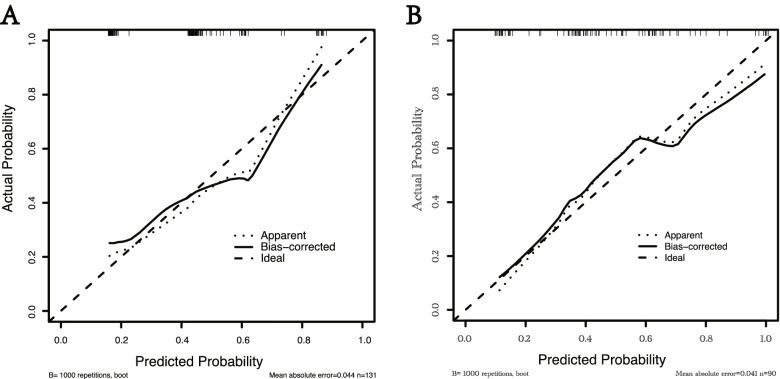
Calibration analysis for VER model in the development cohort (**A**) and the validation cohort (**B**). Calibration analysis displayed a favorable agreement between the prediction and actual outcome of VER in both cohorts

**Fig. 6:**
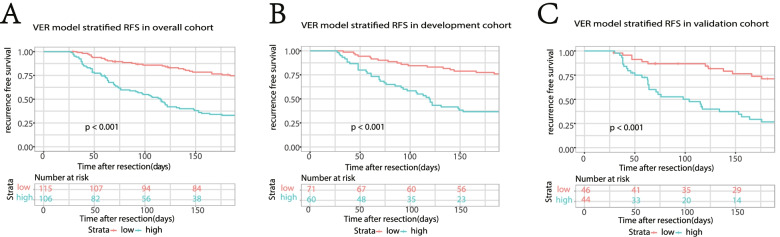
Survival analyses using VER model for patients in overall cohort (**A**), development cohort (**B**) and validation cohort (**C**), in which red line represents RFS of patients at predicted high-risk of VER and green line represents RFS of patients at predicted low-risk of VER. (*P* < 0.001). Our VER model is capable of stratifying patients into two risk groups with significant recurrence outcome

The C-indexes of the prediction of the VER model in development cohort and validation cohort were 0.77(95% CI: 0.69–0.85) and 0.76 (95% CI: 0.66–0.86), while those of AJCC 8th staging was 0.64(95% CI: 0.57–0.72) and 0.64(95% CI: 0.54–0.74). *P* value less than 0.05 showed the significant difference, which signified the superiority of VER model than AJCC 8th staging (Fig. 3A, B). In addition, the VER model showed a better net benefit than AJCC 8th staging in both cohorts (Fig. 4A, B). Calibration analysis also displayed a favorable agreement between the prediction and actual outcome of VER in both cohorts (Fig. 5A, B). Using a 50th centile of the VER risk score, 3.96, in overall cohort, development cohort, and validation cohort, respectively, we stratified the patients into two risk groups with significant recurrence outcome (*P* < 0.001) (Fig. 6A–C).

## Discussion

cHCC is a subtype of primary liver carcinoma composed of HCC and ICC, with a high incidence of recurrence [1–5]. Some researches display that patients with cHCC tend to have recurrence earlier than those with HCC, and similar to those with ICC [2, 3, 9, 10, 24]. Several previous studies reported that more than half of patients with cHCC suffered a dismal prognosis of 2-year early recurrence [4–8]. Similarly, 136 out of 221 patients (61.5%) experienced early recurrence in our study. Therefore, concentrating on ER with a cut-off of 2 years may not be appropriate to the recognition of the recurrence high-risk patients.

In the field of other malignant tumors, previous studies have brought forward the definition of VER and researched risk factors of VER [11–13, 25]. Up to now, no one has proposed the definition of VER in cHCC. On the basis of our multiple center data, 43.4% of patients (96 out of 221) had recurrence within 6 months after the hepatic resection and 67.1% of those suffered the recurrence (96 out of 143) had recurrence within 6 months after the hepatic resection. Meanwhile, several previous studies proposed the definition of VER using a cut-off of 6 months in the research of HCC and ICC [12, 13]. Given that cHCC consists of HCC and ICC, we deem it appropriate to determine the optimal cut-off of VER in cHCC as 6 months like most of the researches of HCC and ICC [11–13]. In addition, several researches demonstrated that patients with PLC who had VER suffered a significant lower OS than those who did not have VER [11, 13].

As such, this study concentrated on the recurrence happened within 6 months after the hepatic resection for patients with cHCC. We identified the independent risk factors of VER and constructed a prediction model integrating MiVI, MaVI, and CA19-9 > = 25 U/mL. Using the VER model, we stratified all patients into two groups with significantly discrete risk of VER (*P* < 0.001). The high risk group consisting of 48.0% of all patients accounts for 71.9% of VER (Fig. 6A). The recognition of recurrence high-risk patients is of vital importance, because the clinician could suggest more vigorous surveillance strategy or appropriate anti-tumor strategies to them. To the best of our knowledge, our study is the first to define and predict VER in cHCC after hepatic resection. An easy-to-use website calculator and a nomogram were provided to facilitate the clinical use.

The clinicopathological features of cHCC resemble those of HCC and ICC [3–5]. There is no existing predictive staging system that is commonly used for cHCC and applying the staging systems for HCC or ICC to cHCC may be problematic. From this perspective, we need a model to predict the recurrence or even VER for patients with cHCC. AJCC staging system is one of the traditional systems applied to the prediction of recurrence in primary liver carcinoma [20], many previous studies have demonstrated the predictive value of AJCC staging [26, 27]. Nevertheless, ROC curves displayed the superior accuracy of our VER model than AJCC 8th staging according to higher C-indexes (0.77 vs 0.64 in development cohort (*P* = 0.012), 0.76 vs 0.64 in validation cohort (*P* = 0.030)) (Fig. 3A, B). Coincidentally, DCA analyze showed that our VER model provided obvious superior net benefit than AJCC 8th staging (Fig. 4A, B). Therefore, our VER model is more practical and more powerful than AJCC 8th staging in clinical use. In addition, the calibration analyze of our VER model also displayed favorable outcome.

Due to the rarity of cHCC and the variety of definitions and pathological types of cHCC, the research on cHCC and its prognosis has been tough and scant. Previous researches demonstrated that the independent risk factors for the recurrence of cHCC include tumor recurrence, tumor size ,metastases, age, MiVI, MaVI, SN, regional organ invasion , elevated CA19–9, ALP, and CEA as well as GGT [1–5]. A limited number of researches identified independent risk factors for the early recurrence of cHCC as follows: tumor size, tumor number, MiVI, MaVI, SN, lymph node metastasis, Midkine, DCP, CA19–9, and poor differentiation [4–6, 28]. Additionally, some research suggest that because cHCC consists of mixed elements of both HCC and ICC, the risk factors for both HCC and ICC would be those for cHCC, which signifies the predictive value of the portion of HCC and ICC [9, 29, 30]. Multi-variable logistic regression analysis identified MiVI, MaVI, and CA199 as independent factors for VER in our study, which is generally consistent with the previous reports.

According to the studies in HCC, MiVI, MaVI, and SN, the HCC related features, are related to the invasion behavior of HCC [17–19, 31–33]. It is widely acknowledged that MiVI, MaVI, and SN are independent risk factors for intrahepatic metastasis, recurrence and survival in HCC [17–19, 31–33]. In the studies in cHCC [4, 28, 34, 35], some previous researches also demonstrated the relationship between MiVI, MaVI, SN, and RFS, which is consistent with the studies in HCC. However, this view still remains controversial because some researches did not identified MiVI, MaVI, and SN as independent risk factors in their analysis [5, 6, 29, 36]. The cause of this controversy can be listed as follows: firstly MiVI, MaVI, and SN were proposed and deeply studied in HCC, actually the pathological features of MiVI, MaVI, and SN in cHCC have not yet been completely investigated and the definitions have not been formulated neither. Unlike tumor size, tumor number, and serum tumor markers (AFP, DCP, and CA19-9), which are readily available and quantifiable, the definitions of MiVI, MaVI, and SN are various or unmentioned in the researches of cHCC, and most researchers tend to define MiVI, MaVI, and SN in cHCC following the definitions in HCC. Secondly, the number of studies in the recurrence of cHCC is limited due to its own rarity and wide variety of pathological subtypes. Our research emphasized the predictive value of MiVI, MaVI for VER in cHCC, thus promoting further researches to determine the pathological features and definitions of MiVI and MaVI in cHCC.

Previous studies elucidated that the elevation of CA19-9 reflects extensive tumor burden, which signifies biologically aggressive behavior, poor differentiation and greater tumor volume of ICC, and correlates with bleak prognosis in ICC [29, 37, 38]. Consistent with this conclusion, some researchers declared the elevation of CA19-9 to be an independent risk factor for RFS and OS in cHCC [5, 29]. It is well known that CA19-9 is a biomarker for ICC components and the prognosis of ICC is worse than that of HCC [2, 37, 38]. Ideally, more portion of ICC lead to worse prognosis for cHCC patients, i.e., ICC dominance (defined as ICC components more than 50% or 80% pathologically according to different studies) is an independent risk factor for recurrence and survival in cHCC. However, some researchers identified that no relationship was found between ICC dominance and the poor prognosis in cHCC [5, 39]. Unfortunately, due to the lack of clinical data, we did not include this variable. More researches are needed to elucidate the relationship between ICC dominance and prognosis in cHCC.

Additionally, people developed VER tend to undergo major resection or lymph node dissection (Table 2), while logistics analysis showed no correlation between the performance of major resection or lymph node dissection and VER (Table 3). Consistent with our research in cHCC, some studies show that the performance of minor resection seems to improve the prognosis in patients with HCC, compared to that of major resection [15, 40], because major resection may influence the postoperative liver function. But no statistically significant difference was found in both DFS and OS in patients underwent major or minor resection [15, 40]. Meanwhile, some researchers recommend lymph node dissection for patients with cHCC, due to the high probability of lymph node metastasis. But no reliable research has been carried out to support the perspective [29, 41]. Compared to HCC and ICC, management of cHCC is not yet standardized, and the choice of surgical procedure still remains controversial. More large-sample research were needed to standardize the treatment of cHCC.

There are three main limitations to our study. First, the clinical data were collected from two centers retrospectively and the sample size is limited, so the information bias, heterogeneity in patients’ characteristics, and different surgical levels between two centers should be taken into consideration. Previous studies identified some variables, AFP, DCP, ALP, tumor size, and tumor number, as independent risk factors for the recurrence of cHCC. Nevertheless, these variables of which the *P* value is small in univariable or multi-variable analysis were identified uncorrelated with VER of cHCC in other research. We need more multi-institutional, large sample-sized, and prospective studies to verify the conclusion. Second, we only enrolled patients from China. Nearly 85% of the enrolled patients had been infected by HBV while few of them had HCV infection or NAFLD. We also excluded those who did not received curative hepatic resection or prior anti-tumor treatments. So, the generalizability of the conclusion is limited. Third, due to the lack of clinical data, we did not include the survival data or the variables which were missing over 10%, such as HCC- or ICC-dominance.

## Conclusions

Nearly half of patients underwent curative hepatic resection for cHCC experienced VER, resulting in a dismal prognosis. This study identified the presence of MiVI, MaVI, and CA19-9 > 25 mAU/mL as the independent risk factors for VER in cHCC patients and constructed a nomogram and an easy-to-use online calculator (https://chcrecurrence.shinyapps.io/myCHCVER2) to help recognize the VER high-risk patients who may be suggested more vigorous surveillance strategy or appropriate anti-tumor strategies.

## Data Availability

The data that support the findings of this study are available from the corresponding author upon reasonable request.
